# Unraveling Torsional Preferences: Comparative Analysis of Torsion Motif Torsional-Angle Distributions across Different Environments

**DOI:** 10.1021/acs.jcim.5c02311

**Published:** 2025-12-09

**Authors:** Jessica Braun, Paul Katzberger, Gregory A. Landrum, Djahan Lamei, Sereina Riniker

**Affiliations:** Department of Chemistry and Applied Biosciences, ETH Zurich, Vladimir-Prelog-Weg 2, Zurich 8093, Switzerland

## Abstract

Understanding the conformational ensemble of molecules in different environments is at the core of many research efforts. In conformer generation and geometry optimization, the complexity of the conformer space arises from the underlying torsion-angle distributions, which, in the case of force fields and some *in silico* conformer generators like ETKDG, are derived from accumulated torsion profiles for a predefined set of torsion motifs (termed ″torsion motif torsional-angle distributions″, tmTADs). Comparative studies of conformer generation and global optimization algorithms often neglect that tmTADs are sensitive to the environment they are extracted from, leading to comparisons of conformational ensembles and minimum-energy conformations from, e.g., crystal versus vacuum environments. Here, we present a large-scale comparative study of tmTADs across different environments, namely crystal, vacuum, water, and hexane, where the ensembles in the noncrystal environments are accessed through a computational workflow using the OpenFF-2.0.0 force field in combination with the graph neural network-based implicit solvent (GNNIS) approach. Our results show that the effects in the different environments, such as solvent–solute interactions in water and hexane, and packing effects in the crystal, produce strikingly distinct torsion distributions for most of the selected torsion motifs. In addition to qualitative and quantitative comparison of the extracted tmTADs, we also provide an automated fitting procedure that allows rapid parametrization of the distributions. These newly found parameters can be employed in a solvent-specific conformer generation procedure in the future.

## Graphical abstract

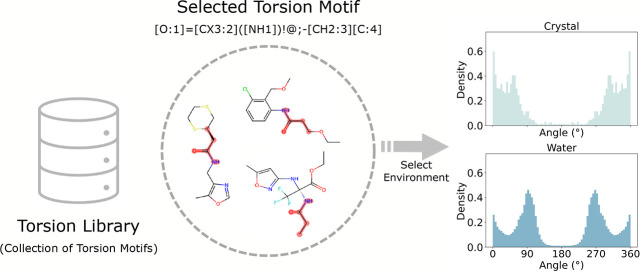

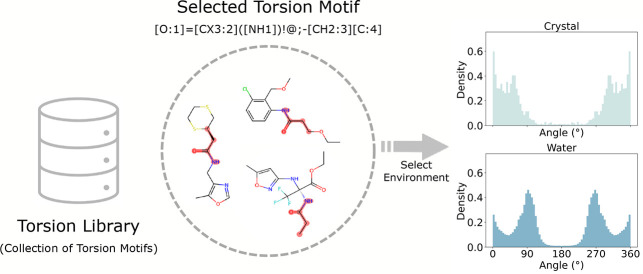

## Introduction

Molecules are three-dimensional (3D) entities, which adapt their conformational behavior according to the surrounding environment. In a crystal environment, the conformational space they occupy is reduced to a small number of conformations, whereas a larger conformational ensemble is found when solvated in any solvent (at *T* > 0 *K*). The conformational ensemble of a molecule is at the core of our understanding of molecular properties, e.g., passive membrane permeability^1,2^ or the likelihood of a molecule to crystallize.^3,4^ The different properties of solvents, for example their respective polarity as expressed by their dielectric constant ϵ_*r*_ or their hydrogen-bonding ability, determine how the solvent and solute molecules interact and, as a consequence, may influence the conformational ensemble of the solute substantially. This can be observed experimentally through nuclear magnetic resonance (NMR),^5,6^ infrared (IR),^7^ and vibrational circular dichroism (VCD) spectroscopy.^8^

Accessing the conformational ensembles with computational methods is the main goal of many research efforts ranging from molecular dynamics (MD) approaches to global optimization methods on the potential-energy surface to *in silico* conformer generation approaches. In 2015, Riniker and Landrum^9^ introduced the ETKDG (experimental torsion basic knowledge distance geometry) conformer generator, which became the default conformer generator in the RDKit,^45^ one of the most widely used cheminformatics toolkits.^10^ Although distance geometry (DG) on its own is a computationally efficient approach to generate diverse conformer ensembles, the strategy to combine it with experimental torsional-angle preferences from small-molecule crystallographic data accelerates convergence to known crystal structures in conformer generation while retaining good sampling of the conformer space.

The initial idea to extract torsion preferences based on a defined collection of molecules originates from Klebe et al.^11^ In 2013, Schärfer et al.^12^ published the first version of the Torsion Library by identifying torsion angles using SMARTS patterns and extracting experimental distributions from the Cambridge Structural Database (CSD).^13,14^ Note that the Torsion Library is exclusively for acyclic single bonds. This Torsion Library was taken as basis for the first version of ET terms in ETKDG.^9^ After an update in 2016 ^15^ (which corresponds to the second version of ET terms in ETKDG), the Torsion Library underwent a major rework in 2022 by Penner et al.^16^ The main part of the 2022 revision focused on automating correctness checking of the SMARTS pattern hierarchy—an important improvement to achieve clean and accurate torsion distributions in the extraction step. Note that the torsion distributions derived from crystal data using the Torsion Library do include some inherent biases, as pointed out by the authors.^16^ These arise from limitations of the crystal data available, an undersampling of high-energy conformations,^17^ and the grouping of the molecules through the SMARTS patterns.

As the introduced biasing terms in the ETKDG conformer generator are derived to yield conformers resembling crystal structures, ensembles obtained through the current ETKDG conformer generation approach might not be suitable for other environments. The fact that ETKDG is optimized to reproduce crystal structures differentiates it from other conformer generators/global optimization approaches such as CREST,^18^ CONFAB^19^ or GOAT.^20^ In practice,^20,21^ conformer generators are often used irrespectively of their originally envisioned application (e.g., crystal structures for ETKDG, or vacuum structures for CREST or GOAT), giving rise to a mismatch between the generated conformational ensembles and the desired environment. To investigate how strongly the environment may affect conformational ensembles and to illustrate how important it is to take the intended purpose of a conformer generator into account when applying it, we designed a large-scale study of torsion profiles in a selection of different environments: crystal, vacuum, water, and hexane. Vacuum and the two solvents were chosen with the following rational: Comparison of vacuum/hexane versus water shows the effect of polarity, whereas the comparison of vacuum versus hexane provides insights into the effect of crowding in solution at similar dielectric permittivity.

The main data set used in this work is a curated subset of the CSD. For the crystal torsion preferences, we extracted torsion profiles using an adapted version of the latest release of the Torsion Library^12,15,16^ directly from the observed crystal geometries. For the vacuum environment, KDG^9^ ensembles were minimized with the OpenFF-2.0.0 force field.^22^ For the water and hexane environments, the initial KDG ensembles were energy-minimized with the OpenFF-2.0.0 force field in combination with the GNNIS (graph neural network-based implicit solvent) model.^23^ With these data sets, we conducted the torsional-angle extractions in the same manner as for the crystal environment before starting our thorough analysis of the differences in torsion preferences. While the computed approach includes possible force-fields effects, which cannot be fully decoupled from environment effects, the comparison of all four environments allows us to (partially) disentangle some of the effects and draw interesting conclusions.

## Methods

### Data Sets

CSD 2024.03 ^13,14^ was used for this study. CSD entries including more than one molecule were split into multiple entries, each containing a single molecule. Any missing hydrogen atoms were added to the molecules using the RDKit’s AddHs­() function. All molecules that included elements other than H, C, N, O, F, Cl, Br, I, S, and P were removed. Furthermore, entries were removed if they did not include at least one carbon–carbon bond. The resulting set included 471’109 3D structures of 321’732 unique molecules.

The second set of molecules used was the same as in ref 24, which is a subset of 370’508 molecules of the DASH data set.^25^ Lehner et al.^25^ designed the DASH data set to provide a collection of lead-like ChEMBL^26^ molecules containing the broadest possible coverage of atom environments defined by bits from Morgan fingerprints with radius = 2. A detailed description of the design and composition of the DASH data set is given in ref 25. It is freely available from the ETH Research Collection (https://www.research-collection.ethz.ch/handle/20.500.11850/667722).

### Torsion Library

For this work, the most recent version of the Torsion Library^16^ was translated into an ordered list that preserved the original hierarchy. The SMARTS were modified to require that the central bond be a single bond in addition to not being a ring bond. SMARTS including ″N_lp″ were adapted as their parsing otherwise relies on a SMARTS extension. Furthermore, 55 patterns were excluded for one of the following reasons:

- Too few matches (<50) in the CSD data set to allow statistically relevant comparisons between profiles from different environments.
- Only three atoms were defined via a specific atom map in the pattern.

The remaining 458 patterns were split into seven hierarchy classes following the original Torsion Library classification based on the elements of the central bond: C–C (167), C–O (52), C–S (15), N–C (118), S–N (14), S–S (1), and G–G (91), where G stands for ″general″. The numbers in parentheses indicate the number of patterns within the respective class. The complete list of the SMARTS patterns used is available in the Supporting Information. A special case is the hierarchy class GG, whose general patterns act as a fallback should a substructure not match a more specific SMARTS in another dedicated hierarchy class. For the analysis, the GG hierarchy class was excluded, as its resulting distributions are more likely to reflect a superposition of multiple underlying torsion profiles, making it less useful for conformer generation. This left 367 patterns for the more in-depth analysis.

### Profile Extraction

The torsion motif torsional-angle distributions (tmTADs) were either extracted from an experimental source (CSD) or derived from conformational ensembles obtained through a computational workflow described in the subsection “Conformer Generation and Minimization Procedure” below. They were generated by performing substructure searches in each molecule to identify bonds corresponding to the SMARTS patterns defined in the Torsion Library and recording the observed torsion angles. The searches used the ordering of the Torsion Library so that each bond can only match a single torsion pattern.

In certain cases, a specific pattern may match a central bond and its neighboring atoms in multiple ways. When this occurs, the observed torsional angles for all possible matches are recorded. This ensures that all observable torsional angles for a matching dihedral are captured rather than a single representative picked at random.

It is common to assume that the accumulated torsion profiles like those considered here are symmetric and to model them with symmetric functions (e.g., sums of cosines).^9,27^ In the following, we briefly outline the rationale for expecting symmetric torsion distributions and describe how our extraction procedure takes this into account.

The torsion definitions expressed in the form of SMARTS patterns do not take stereochemistry into account, thus the pattern that matches atoms 1–2–3–4 in Figure 1A will also match atoms 1–2–3–4 in its mirror image (Figure 1B). Given a crystal structure of stereoisomer A where the torsion 1–2–3–4 is equal to 10°, taking the mirror image of the entire crystal structure yields stereoisomer B and torsion 1–2–3–4 equal to 350° (or −10°). This mirrored crystal structure would have exactly the same energy (along with all other nonchiral properties). Although it was not observed, there is no reason to assume that this structure cannot exist; hence, we update the distribution for the torsion pattern that matches atoms 1–2–3–4 with both 10° and 350°.

**Figure 1:**
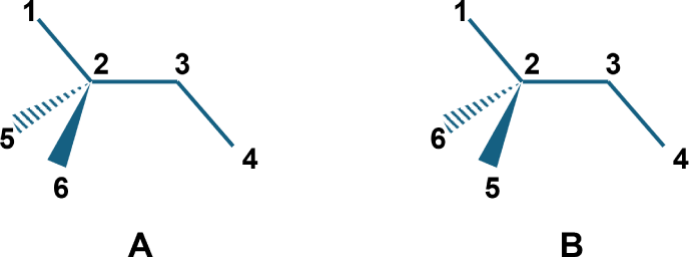
Illustration of possible atom arrangement around a dihedral matching a SMARTS pattern in a pair of enantiomers. (A): Example molecule. (B): Mirror image of the example molecule where the mirror plane is defined by atoms 1, 2, and 3.

All profiles in this paper show the data obtained through the described data-augmentation approach.

### Fitting tmTADs with Wrapped Mirrored Gaussians

We developed an automated fitting procedure to describe the experimental tmTADs. tmTADs originating from accumulated profiles of multiple molecules feature a periodicity of 2π and a symmetry around zero or π depending on the chosen angle domain. Given these characteristics, the selection of potential functionals is limited, making a truncated Fourier series a common choice (see, e.g., refs 9, 27 )­

1 $$V\left(\phi \right)=\overset{M}{\underset{i=1}{\sum }}K_{i}\left[1+s_{i}\cos\left(i\phi \right)\right]$$

where *s*_*i*_ ∈ {−1,1} is the phase shift, *K*_*i*_ the force constant, and *M* is the highest possible multiplicity. The set value for *M* varies between three, four, and six depending on the force field.^9^

Here, a new fitting procedure is introduced using wrapped mirrored Gaussians. We assume that any asymmetries in the extracted torsion distributions from experimental sources are due to limited data availability. As we did not want the fitted functions to reflect these biases, we mirrored each experimental distribution at π and then applied a 1D Gaussian filter to smooth the result. The histogram was then partitioned into different regions with a peak detection algorithm based on SciPy’s^28^ find_peaks functionality. The SciPy algorithm was adjusted to account for the periodicity (details in Section 2.1 in the Supporting Information). We limited the number of peaks/regions to, at most, three in the interval [0,π]. This corresponds to a maximum multiplicity of six for each torsion profile.

Within each region, the distribution was modeled by a wrapped Gaussian (related to the wrapped normal distribution^29^),

2 $$P_{i}\left(x\right)=a_{i}\cdot \exp\left(-{\left(\frac{\left(x-b_{i}+\pi \right)\mathrm{mod}2\pi -\pi }{c_{i}}\right)}^{2}\right)$$

with the *b*_*i*_ parameters set to be the locations of the peaks. The triplets of (*a*_*i*_, *b*_*i*_, *c*_*i*_) parameters identified in this manner were used in the following functional form to describe the torsion profile,

3 $$\begin{array}{ll} P\left(x\right) & =\overset{3}{\underset{i=1}{\sum }}a_{i}(\exp\left[-{\left(\frac{\left(\left(x-b_{i}+\pi \right)\mathrm{mod}2\pi \right)-\pi }{c_{i}}\right)}^{2}\right]\\ & +\exp\left[-{\left(\frac{\left(\left(\left(2\pi -x\right)-b_{i}+\pi \right)\mathrm{mod}2\pi \right)-\pi }{c_{i}}\right)}^{2}\right]) \end{array}$$

The *a*_*i*_ and *c*_*i*_ were fitted by minimizing the error of the fit against the smoothed histogram across the entire range [0,2π]. Using eq 3 means that each (*a*_*i*_, *b*_*i*_, *c*_*i*_) triplet describes two peaks at once: The one within the interval [0,π] and its mirror image in [π,2π]. In the case where a peak is located at 0 (meaning also at 2π) and eq 3 results in a direct overlap of the two mirror peaks around π, the *a* parameter was scaled to compensate for the overlay.

As a future application, we envision the fits being used in a conformer generation approach like ETKDG, where they would be used to calculate energies and gradients, which requires the fitted functions to be fully differentiable. As the modulo operator is not fully differentiable in [0,2π], it was replaced by the following function, which agrees pointwise on [0,2π],

4 $$\begin{array}{ll} P\left(x\right) & =\overset{3}{\underset{i=1}{\sum }}a_{i}(\exp\left[{-\left(\frac{x-b_{i}}{c_{i}}\right)}^{2}\right]+\exp\left[{-\left(\frac{2\pi -x-b_{i}}{c_{i}}\right)}^{2}\right]\\ & +\exp\left[{-\left(\frac{x-b_{i}+2\pi }{c_{i}}\right)}^{2}\right]+\exp\left[{-\left(\frac{4\pi -x-b_{i}}{c_{i}}\right)}^{2}\right]) \end{array}$$

Alternatively, the distribution can be modeled using von Mises functions, as demonstrated by Friedrich et al.^30^

### Conformer Generation and Minimization Procedure

In order to generate conformational ensembles for each molecule, the procedure described in ref. 23 was used (Figure 2). An initial KDG ensemble of 100 conformers was created using the RDKit (version 2024.09.1),^45^ and subsequently minimized using OpenMM 8.1.2 ^31^ with the OpenFF-2.0.0 force field and the GNNIS solvation model. For each ensemble, all coordinates, energies, and extracted tmTADs for the relevant dihedrals were stored. Note that this workflow does not aim to exhaustively cover the conformational space for each molecule (which would require a much larger number of conformers for many of the molecules), but to achieve good sampling of each torsion profile by combining the results from multiple molecules. The results for all conformer ensembles obtained through the GNNIS workflow include the potential energies of the conformers (but not free energies as we did not apply the last step of the workflow in ref 23).

**Figure 2:**
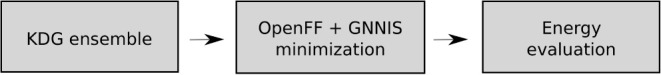
Schematic representation of the energy-minimization workflow starting from a stochastic KDG ensemble. Further details and discussion are given in ref. 23.

For each ensemble, we used the minimum-energy conformer to calculate the relative energies of the others. The influence of the relative-energy threshold on the energy-pruning step is addressed in the Results and Discussion section below. Unless stated otherwise, the energy threshold used in the following analysis was 25 kJ/mol.

### Selection of Environments

To study the impact of different solvents on computed tmTADs, we selected two solvents (from the 39 that GNNIS was trained for^23^) for which we expect different behavior: water (TIP3P) and hexane. Their profiles are compared to the OpenFF-2.0.0 vacuum reference and crystal tmTADs extracted from the CSD.

### Comparison of Torsion Profiles

The pairwise comparison of torsion distributions is a difficult task. Instead of simple sample calculations, e.g., comparing the mean or 95-percentile value, we need to resort to an appropriate measure of statistical distance for our specific problem. In an expert-driven comparison of torsion distributions, the focus is generally placed on the characteristics of the peaks of the distributions. Changes of interest are shifts in peak positions, peak broadness, changes in the relative peak heights, and peak appearances and disappearances. Taking these into account requires a distance metric that is sensitive to both global and local changes (mass and shape) in the distributions. One appropriate metric for this task is the second-order Wasserstein distance *W*_2_,^32,33^

5 $$W_{2}\left(\mu ,\nu \right)=\underset{\begin{array}{l} X∼\mu \\ Y∼\nu \end{array}}{\inf}{\left(E||X-Y|{|}^{2}\right)}^{\left(1/2\right)}$$

where the infimum is taken over all pairs of random vectors *X* marginally distributed as μ and *Y* as ν, respectively.^33^ In contrast to the more generally known first-order Wasserstein distance *W*_1_, which describes the work to transform one distribution into the other, *W*_2_ is proportional to the square of the distance that the mass travels (due to the defined cost function). A short overview over the Wasserstein distances and their differences is provided in Section S3 in the Supporting Information. The calculation of *W*_2_ was carried out with the Python Optimal Transport library.^34^

Another method of estimating the distance between two distributions is the Cramér-von Mises distance,^35^

6 $$\Delta \left(F,F_{0}\right)=\int_{-\infty }^{\infty }{\left(F(x)-F_{0}(x)\right)}^{2}dF_{0}\left(x\right)$$

Anderson^36^ extended the original mathematical formulation, which tested whether a sample was drawn from a specific continuous distribution, to testing whether two samples come from the same distribution, as shown in eq 7,

7 $$N\omega^{2}\approx \frac{NM}{N+M}\int_{-\infty }^{\infty }{\left(F_{N}(x)-G_{M}(x)\right)}^{2}dH_{N+M}\left(x\right)$$

*F*_*N*_(*x*) and *G*_*M*_(*x*) are empirical distribution function of the two samples, respectively. *H*_*N+M*_(*x*) is the empirical distribution function of both samples together. For the calculation of ω^2^, the SciPy implementation^37^ was used. ω^2^, also known as the Cramér-von Mises *criterion* is related but not equal to the Cramér-von Mises *distance*. Here, we used ω as one measure of statistical distance. As eq 7 shows, there is a dependency on *N* and *M*, hence we bootstraped by randomly sampling 10’000 points with replacement from the observed distribution to have comparable distances between all evaluated distributions.

As both statistical distances, *W*_2_ and ω, differ in which features (i.e., peak shift, difference in relative peak height, peak appearances/disappearances) they are most sensitive to, they might disagree in certain cases if distributions are close together or further apart. For a better understanding of the agreements and disagreements, we constructed four example comparisons and the ranking from most similar to most different was compared between ω and *W*_2_. The four scenarios presented in Figure 3 are as follows: The first case (Examples 1 versus 2) shows two random samples drawn from the same distribution. The second case (Examples 3 versus 4) highlights the splitting of a single peak into two distinct peaks. The third case (Examples 5 versus 6) illustrates the broadening of the two existing peaks, whereas the fourth case (Examples 7 versus 8) showcases a reversal in the relative heights of the peaks.

**Figure 3:**
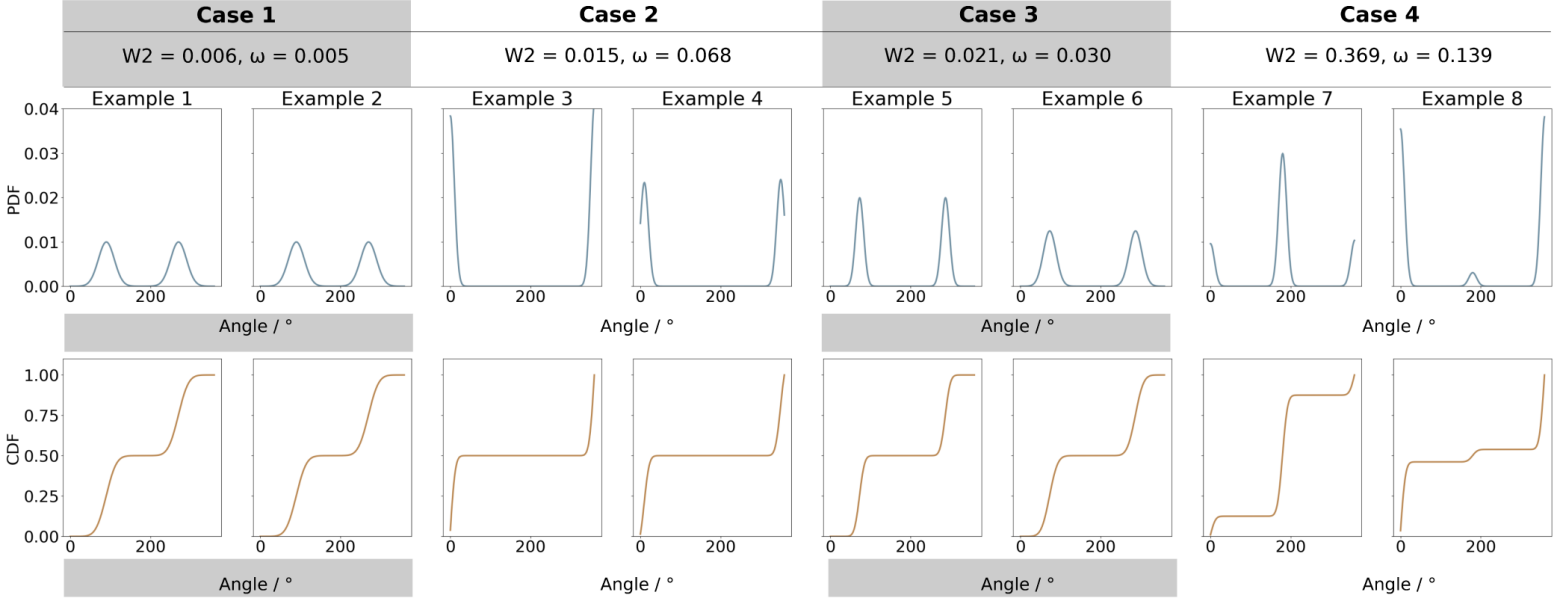
Four cases illustrating the relationship between the statistical distances *W*_2_ and ω, showcasing their differences and agreements in distance estimates. (Top): probability density functions (PDF). (Bottom): cumulative distribution functions (CDF).

Figure 3 also shows the resulting *W*_2_ and ω values for these four test cases. When comparing the ranking based on the two metrics, both agree on the most similar and most different cases (case 1 and 4, respectively), but they rank cases 2 and 3 differently. According to *W*_2_, the distributions in case 2 are more similar than the two in case 3, whereas ω reverses that order. The observed behavior can be explained by the fact that *W*_2_ penalizes local changes in the distribution according to the distance over which the probability mass is moved. If the distance is small, as in the case of a single peak splitting into two peaks, the penalty imposed by *W*_2_ is rather small. In comparison, ω is more sensitive to these local disturbances as reflected in the cumulative distribution functions (CDFs). Both metrics are valid rankings as there is no ground truth when it comes to the distance between two torsion distributions.

Normalization was performed by dividing each metric value by the theoretical maximum value. For details on the derivation of the maximum values see Section S5 in the Supporting Information. For the remainder of this study, all reported ω and *W*_2_ values were normalized. For *W*_2_, we set an empirically defined threshold of 0.03 and for ω of 0.07 to describe two distributions as significantly different from each other.

Table 1 summarizes the pairwise comparisons performed between tmTADs of different environments. The first row includes all comparisons between the crystal environment and the results of our computational workflow, capturing differences arising from both the force field and the solvent contributions from GNNIS. The other comparisons are only within computational results and therefore capture differences arising from the GNNIS contributions (solvent contributions).

**Table 1:** Summary of Comparisons of tmTADs from Different Environments^a^

environment 1	environment 2		
	vacuum	hexane	water
crystal	X	X	X
vacuum		X	X
hexane			X

a X includes all comparisons between the crystal environment and the results of our computational workflow. Underlined X represents comparisons within computational results.

## Results and Discussion

### tmTADs across All Environments

The evaluation of statistical distances between tmTADs is based on the second-order Wasserstein distance *W*_2_ and the Cramér-von Mises metric ω.

Figure 4 shows the correlation between the two metrics for all tmTADs when comparing the crystal environment versus the vacuum environment. As discussed in the Methods section, each metric emphasizes different features of the distributions and penalizes other aspects, which explains the lack of a strong correlation between the two. This is illustrated in Figure 5, which presents the CDFs for the two metrics. A rapid rise of a CDF at small metric values indicates that a large fraction of distribution pairs have low statistical distances, i.e., many similar profiles.

**Figure 4:**
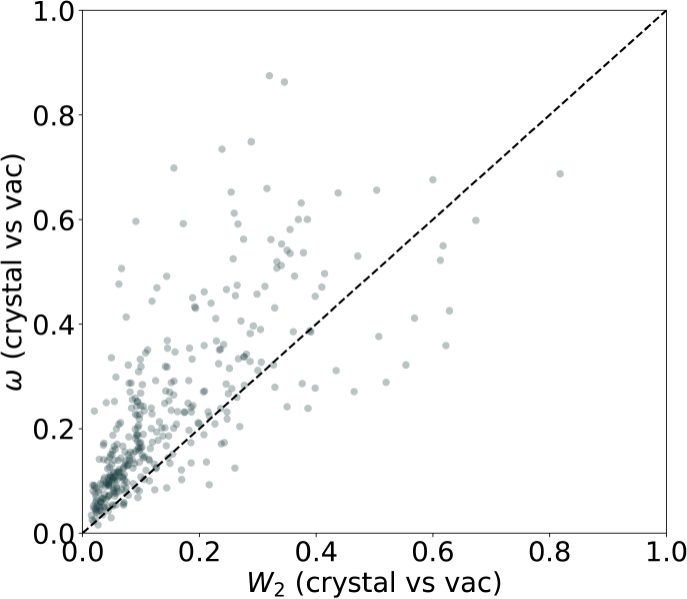
Comparison of all accumulated torsion profiles in the crystal and vacuum environments with the normalized second-order Wasserstein distance *W*_2_ (*x*-axis) and the Cramér-von Mises metric ω (*y*-axis).

**Figure 5:**
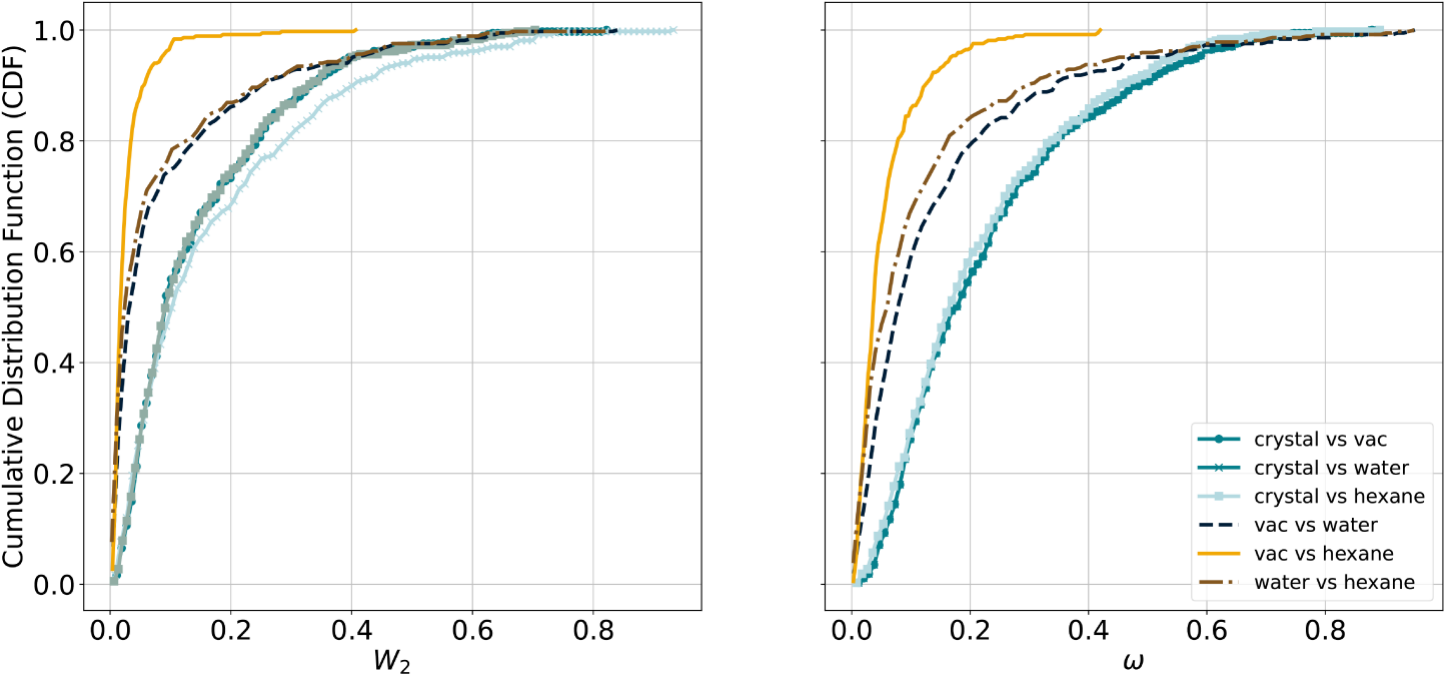
Cumulative distribution functions (CDFs) of the two metrics *W*_2_ and ω for all six comparisons (see legend and Table 1).

Both metrics display consistent overall trends. Comparisons between the crystal and any of the other three environments share very similar CDFs across the metrics with only small visible differences and an unchanged ranking. Moreover, the CDFs show that every comparison involving the crystal and another environment yields larger statistical distances, indicating the crystal is the most distinct environment. Not surprisingly, the closest resemblance of torsion profiles, marked by the CDF describing the smallest statistical distances through locality at small *W*_2_ and ω values plus a near-vertical rise, can be found between the vacuum and hexane environments. These trends follow chemical expectations, and are thus a confirmation that our chosen evaluation protocol depicts differences and similarities in a meaningful way.

Furthermore, Figure 5 shows that the torsion profiles from the crystal environment are significantly different from the three others. Three potential explanations for this are (1) differences in the intermolecular interactions between the environments (between molecules in the crystal vs solute–solvent interactions), (2) biases introduced by the force-field parametrization, e.g., torsion potentials, and (3) differences in the sampling of the conformational space (single observed conformer per molecule in the crystal vs an ensemble in all other environments). One way to assess the force-field effect could be to minimize the crystal conformations with the force field. However, if this is done for an isolated conformer, the environment is not correct (vacuum instead of crystal), and if it is done for the periodic unit cell, the crystal packing prohibits larger conformational changes during energy minimization (see Section S9 in the Supporting Information). A better evaluation of the force-field preferences in the crystal environment would require crystal-structure prediction using that force field, which is outside of the scope of this study. Thus, we conclude that one or more of these explanations will be true for any of the 367 tmTADs.

Importantly, the differences between the tmTADs indicate that torsional preferences are specific for the environment they were derived from and should in most cases not be used for another environment.

### Population Analysis of the SMARTS Patterns

It should be noted that the number of data points per SMARTS pattern can vary greatly with the different population numbers also mirroring natural biases when analyzing the CSD data set. Table 2 gives the numbers of matches for the DASH and CSD data sets over the entire torsion hierarchy per hierarchy class (bond type).

**Table 2:** Number of Molecules that Matched SMARTS Pattern Hierarchy Classes in the Two Data Sets, CSD and DASH^a^

Hierarchy class	CSD data set	DASH data set
C–C	243573 (46)	323347 (43)
C–O	122775 (23)	136486 (18)
C–S	33471 (6)	47770 (6)
N–C	115725 (22)	223247 (30)
S–N	13374 (3)	19612 (3)
S–S	714 (0.1)	234 (0.03)

a In parentheses are the percentages of molecules in the different hierarchy classes .

### Influence of the Chosen Data Set

When comparing torsion distributions derived from different molecules within the same environment, discrepancies, particularly in features such as relative peak heights, may simply reflect biases introduced by the selection of molecules in the data set. Figure 6 shows the metrics *W*_2_ and ω for all comparisons of torsion distributions with enough sampling (at least 300 data points each) in the water environment between the CSD and DASH data sets. While most tmTADs are highly similar between the two data sets, there are distinct outliers arising because the CSD and DASH sets are covering partially different chemical spaces. After inspecting the outliers, several reasons for their existence were identified. Besides the differing coverage of the chemical space, we found that the preparation of the two data sets regarding structural isomers (i.e., whether or not some functional groups are represented in their zwitterionic forms) was responsible for large differences. This is further discussed, with an example, in Section S6 in the Supporting Information.

**Figure 6:**
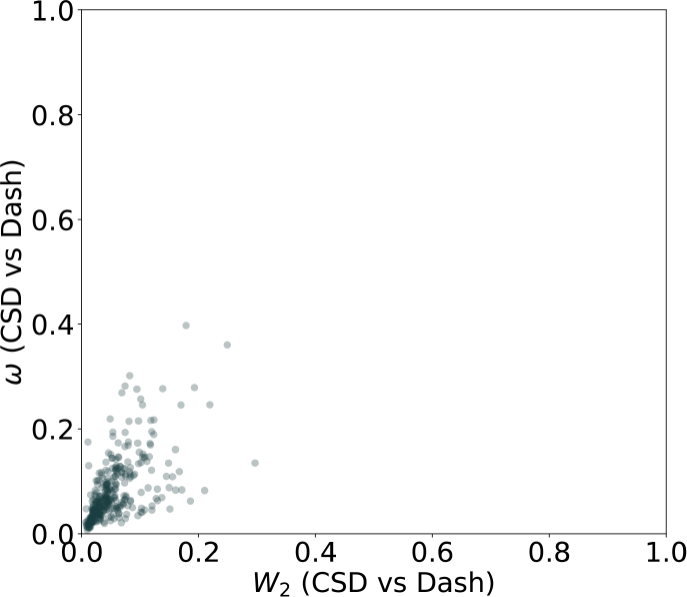
Comparison of all accumulated torsion profiles in the water environment for the DASH and CSD data sets with *W*_2_ and ω.

It is important for the future application of tmTADs to understand that their exact shape can depend on the selected data set. However, relative comparisons between torsion profiles from the same set across different environments—and the conclusions drawn from these comparisons—are independent of such an analysis and not significantly influenced by the data set used.

### Influence of the Energy Cutoff during the Ensemble Processing

The choice of energy cutoff (see subsection Conformer Generation and Minimization Procedure) has a noticeable impact on the overall shape of the torsion profiles. In most cases, varying the energy cutoff altered the relative heights of the peaks within a profile, while the positions of the peaks remained largely unchanged.

The effect is illustrated for the example [CX3H0:1]=[CX3H0:2]!@;-[CX3:3]=[CX3:4] in Figure 7 for the vacuum environment. In this case, reducing *E*_cutoff_ from 50 to 25 kJ/mol does not substantially alter the overall profile shapes. However, lowering the cutoff to 5 kJ/mol leads to a pronounced decrease in the population of the peaks near 40° and 320°. The same behavior is observed in the water and hexane environments, as detailed in Section S8 of the Supporting Information. As slightly higher-energy conformations are still of interest in the further application of the profiles, the threshold of 25 kJ/mol was chosen for all conducted analysis.

**Figure 7:**
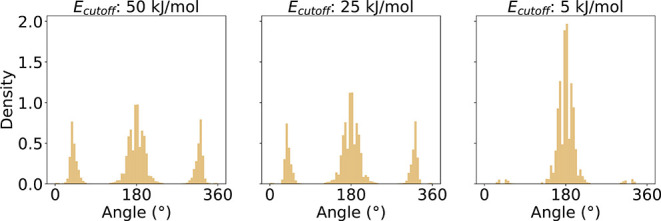
tmTADs for [CX3H0:1]=[CX3H0:2]!@;-[CX3:3]=[CX3:4] in vacuum for three different energy cutoffs in the ensemble processing.

### Observed Crystal Packing Effects

Although a comprehensive explanation of the observed differences across all environments is beyond the scope of this study, we present some illustrative case studies. The SMARTS pattern [cH1:1]­[c:2]­(cO)!@;-[O:3]­[C:4] shows striking differences between the different environments (Figure 8). While the torsion distributions in vacuum, water, and hexane closely resemble each other, the crystal environment presents as a clear outlier. All four environments agree on a peak at 0°/360°, but there are no peaks for the crystal environment at 131°/229° and 103°/257°. To rationalize what forces the torsion value of the dihedral identified by the SMARTS pattern to a strict value of 0°/360° in the crystal environment, an example match from the CSD was picked with its molecular graph shown in Figure 9A. In the crystal packing, shown in Figure 9B, the preference for the torsion at 0°/360° can be explained by it accommodating the intermolecular hydrogen bond formed between molecular copies.

**Figure 8:**
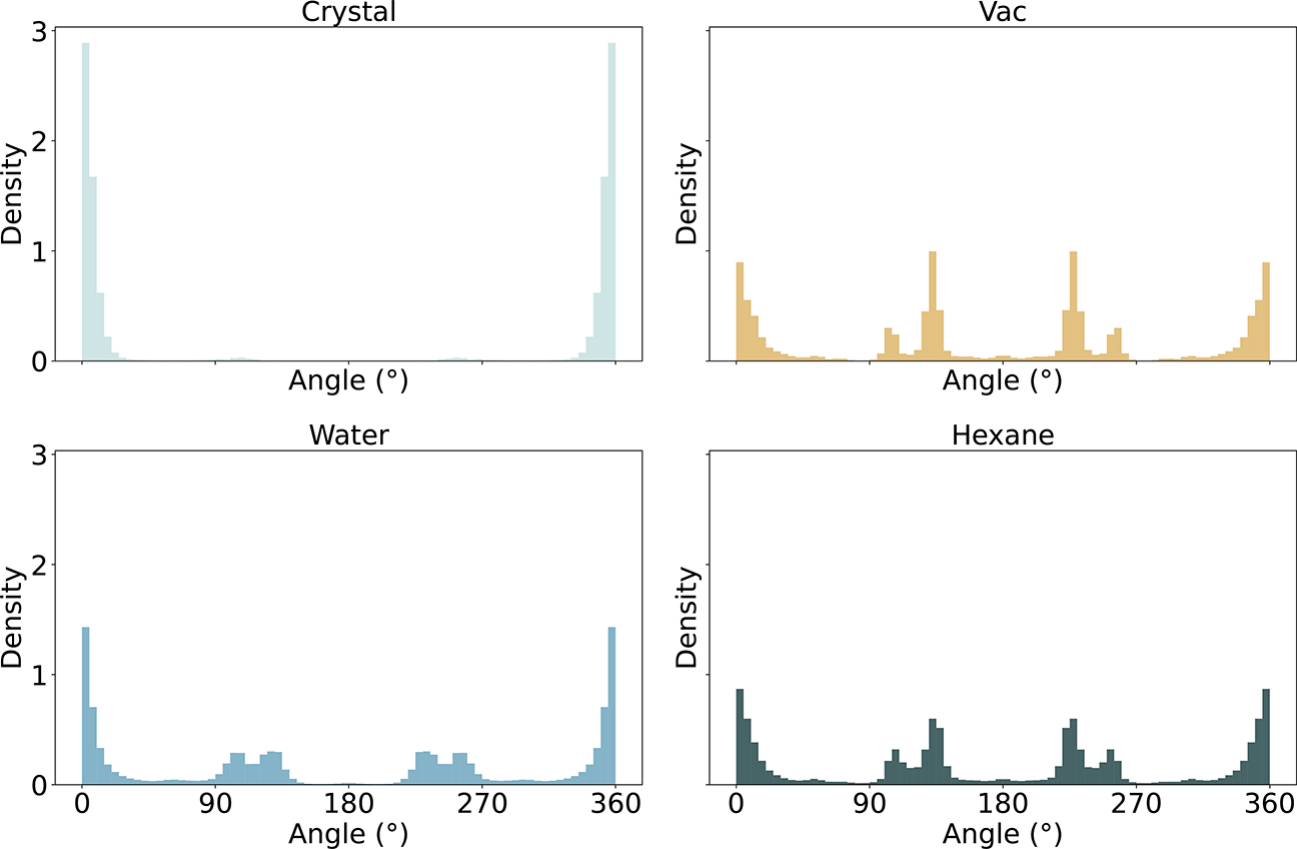
Torsion distributions for SMARTS pattern [cH1:1]­[c:2]­(cO)!@;-[O:3]­[C:4] in the selected environments. For vacuum, water, and hexane, the profiles display all torsion values for conformers extracted at an energy threshold of 25 kJ/mol.

**Figure 9:**
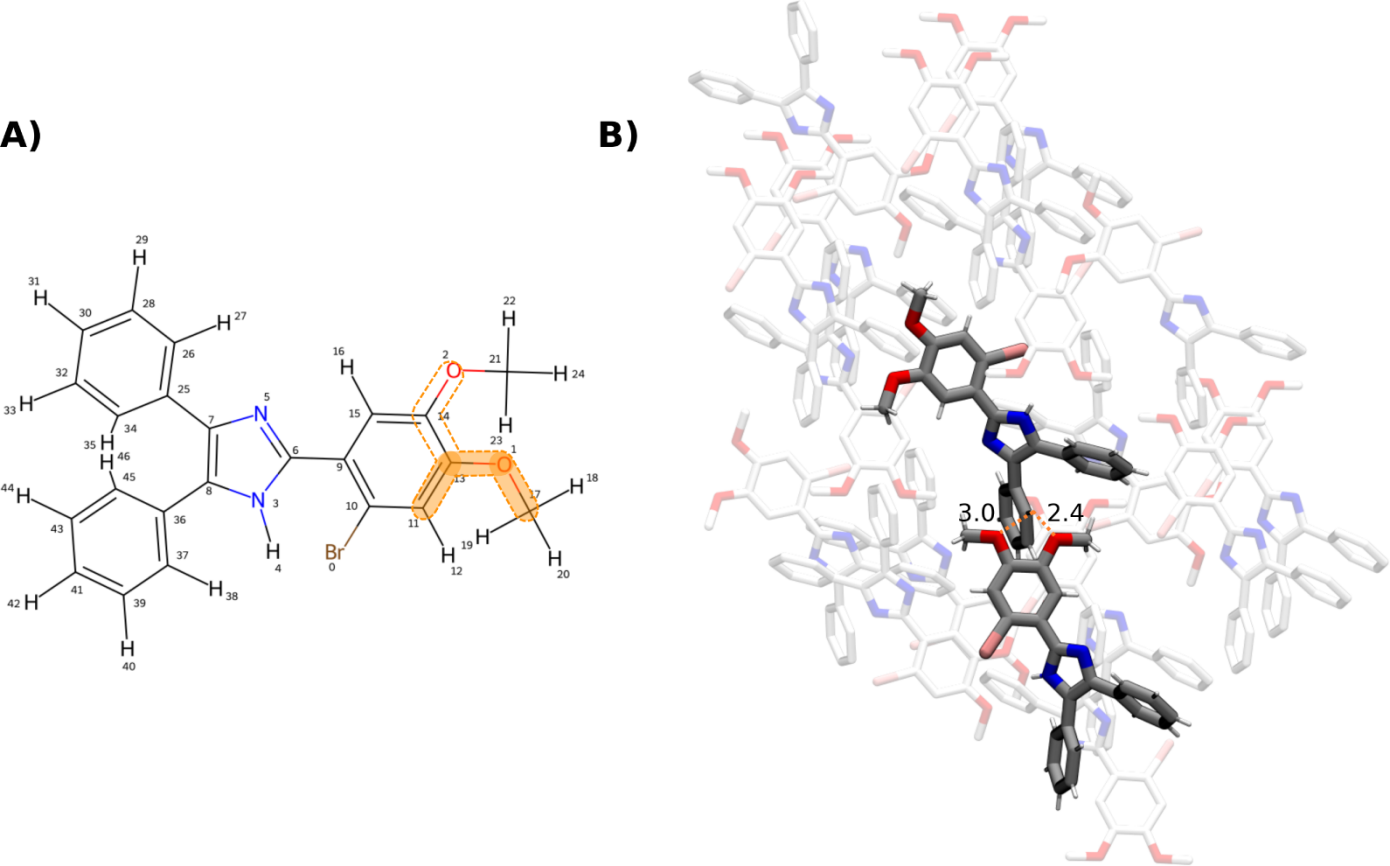
(A): Example molecule (from the CSD data set). Atom matches to the SMARTS pattern [cH1:1]­[c:2]­(cO)!@;-[O:3]­[C:4] are marked by the dashed lines. The torsion values are extracted for the dihedral formed by atoms (11,13,1,17), highlighted in orange. (B): Packing of the molecule in the crystal (CSD identifier ABAGUJ^38^). The distance of 2.4 Å from the H atom of one copy to the O atom of another indicates the formation of an intermolecular hydrogen bond.

### Solvent–Solute Interaction in Water

Based on *W*_2_ and ω, a total of 17 cases were identified where the tmTADs of the water and crystal environments resemble each other more closely than water to hexane or vacuum. These cases are of particular interest as the observed differences cannot be fully explained by force-field effects. Figure 10 presents an example for this case with SMARTS pattern [$([cH0]­F):1]­[c:2]­([cH1])!@;-[CX3:3]­([NX3H1])=[O:4]. Driven by conjugation, it is expected that the torsion profile exhibits preferences for 0° and 180^◦^. Due to a steric hindrance (bumping of hydrogen atoms 28 and 29), a displacement at the 0° dihedral configuration is observed.

**Figure 10:**
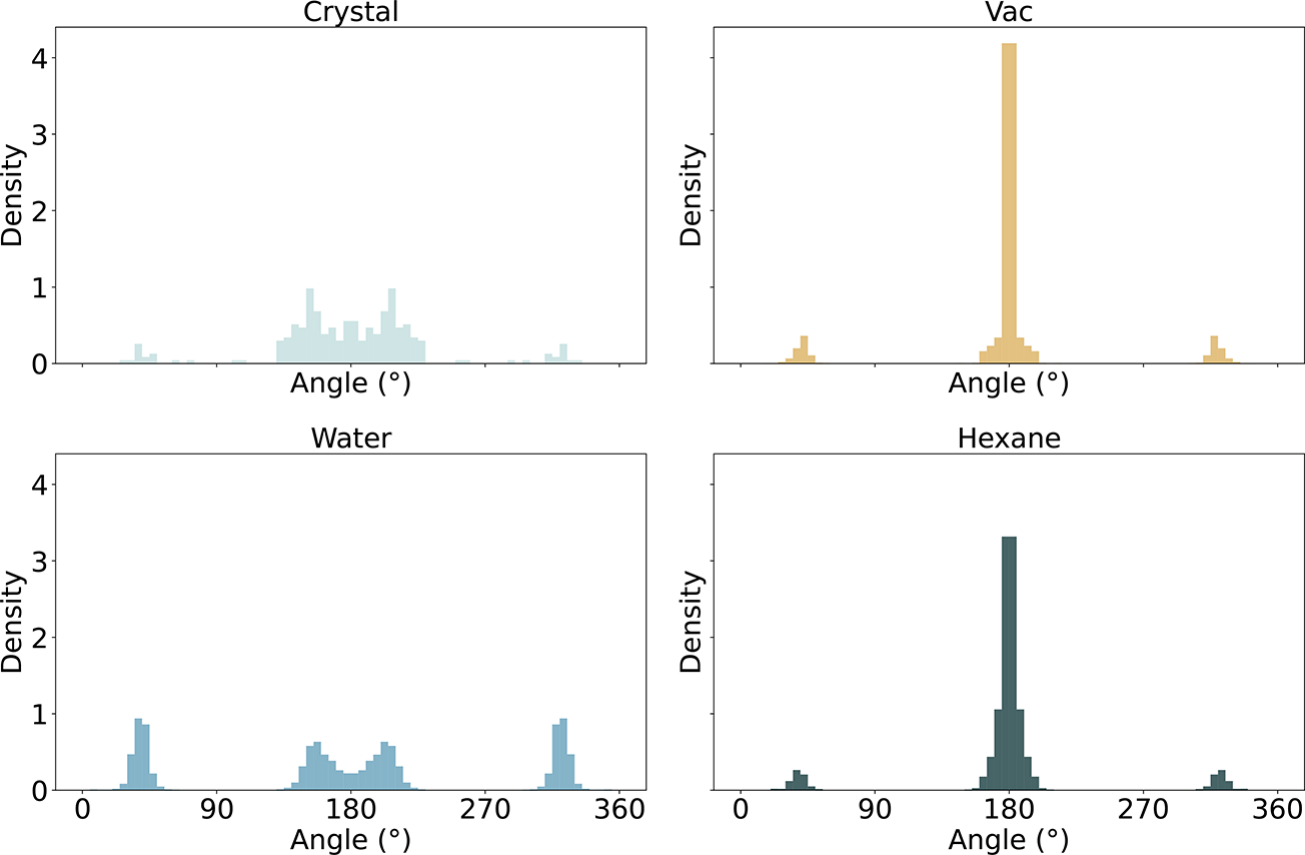
Torsion distributions for SMARTS pattern [$([cH0]­F):1]­[c:2]­([cH1])!@;-[CX3:3]­([NX3H1])=[O:4] in the four environments.

A closer look at one of the molecules matching the SMARTS patterns demonstrates the impact of intramolecular hydrogen bonds on torsion profiles (Figure 11A). Within molecules exhibiting this SMARTS pattern, two potential favorable interactions can form: an interaction between the fluorine atom (atom 1) and the hydrogen (atom 29) attached to the nitrogen (atom 3), and a hydrogen bond between the oxygen atom (atom 6) and the hydrogen (atom 28) bonded to the aromatic ring. At a dihedral torsion angle of 179.5°, the distance between the atoms 1 and 29 (F···H) is 1.9 Å and between atoms 6 and 28 (O···H) 2.5 Å which is in line with the generally assumed requirement for an intramolecular hydrogen bond.^39^ These intramolecular interactions lead to the formation of a pseudo-five and six-membered ring, respectively.

**Figure 11:**
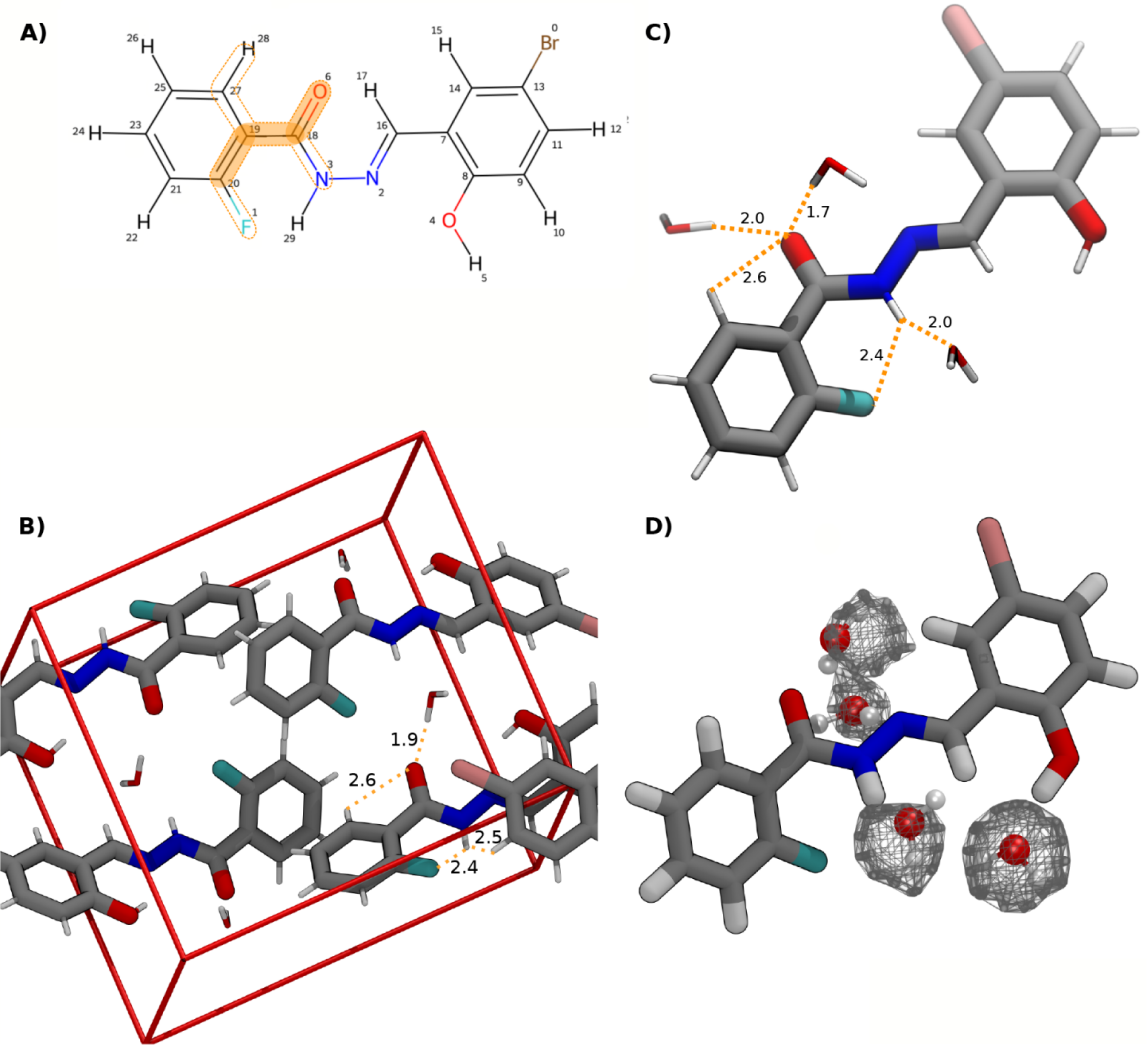
(A): Example molecule (from the CSD data set). The atom matches to the SMARTS pattern [$([cH0]­F):1]­[c:2]­([cH1])!@;-[CX3:3]­([NX3H1])=[O:4] are highlighted by the dashed lines. The torsion values are extracted for the dihedral formed by (20,19,18,6), marked in orange. (B): Packing of the molecule in the crystal (CSD identifier ABOQOZ).^40^ Two intramolecular interactions are in competition with a hydrogen bond formed with a cocrystallized water molecule and a molecular copy forming an interaction. (C): Exemplary snapshot from the MD simulation of the same molecule in coordination with three water molecules forming hydrogen bonds with the solute. The dihedral torsion value of the conformation presented is at 146°. (D): Snapshot of the same MD trajectory as in (B) with average hydration density map calculated with VolMap/VMD using a contour level of 75%.

Note that the deviations from 180° observed in the crystal and water are caused by different factors. In the crystal environment, intramolecular interactions are in competition with intermolecular interactions between copies within the packing structure. As can be seen in Figure 11B, the arrangement of the symmetry equivalents in the unit cell allows for the formation of a hydrogen bond with cocrystallized water, as well as an interaction of the fluorine substituent with hydrogen-bond donors of other copies. The observed O···H distance of 1.9 Å and the F···H distances of 2.4 and 2.5 Å are in line with the value ranges reported in literature.^41^

In the water environment, on the other hand, the intramolecular interactions are in competition with hydrogen bonding with the water molecules. It is difficult to quantify these effects, but a qualitative explanation for the interference of the water molecules can be provided by performing a short MD simulation in water to extract an exemplary solvent configuration (Figure 11C), as well as calculating a hydration density map with VolMap/VMD to confirm that the solvent configuration is representative (Figure 11D). As a starting conformer, a conformation from the water ensemble exhibiting a torsion angle of 163° in the dihedral of interest was selected at random from the two peaks observed below and above 180°. Its atom positions were restrained and a short MD simulation in water was performed. Both intramolecular interactions are still present, but in addition, three hydrogen bonds are formed with nearby solvent molecules. All three of the O···H distances (1.7, 2.0, and 2.0 Å) are below the sum of the van der Waals radii of *r*_*vdW*_(*H*) = 1.2 Å and *r*_*vdW*_(*O*) = 1.5 Å.^42^

## Conclusions and Outlook

We compared the torsion distributions of a set of SMARTS patterns for two molecule data sets (CSD and DASH) between four different environments: crystal, vacuum, hexane solution, and water solution. All noncrystal environment torsional-angle profiles were derived using a computational workflow using the OpenFF-2.0.0 force field and the GNNIS implicit solvent model. In contrast to some previous studies, which either stated that no difference was found^43^ or indirectly assumed that tmTADs from different environments are comparable,^20^ we observed significant differences between a considerable number of the tmTADs in these different environments, which are not caused by undersampling but rather a result of the environment strongly influencing the conformational preferences of molecules. Through case studies, we were able to rationalize observed trends including the higher similarity of torsion profiles in hexane and vacuum than hexane and water. The (experimental) crystal environment showed the largest difference from the other (computed) environments. In light of these findings, we want to encourage the scientific community to pay attention when conducting comparisons and reviews of conformer generations and not compare procedures optimized for different environments. As discussed, all computational tmTADs include a force-field contribution and thus, the chosen force-field parametrization can affect the resulting distributions. Studying similar distributions from QM geometry optimizations with an appropriate implicit solvent model could resolve this issue, but this requires a sufficiently fast method to handle the large number of molecules and conformers. Machine-learning interaction potentials may provide the necessary speed and accuracy, and their exploration for this purpose will be part of future work.

The resulting tmTADs and the introduced functional form of the wrapped mirrored Gaussians are ready to be integrated as environment-specific torsion preferences within conformer generation protocols such as ETKDG.

## Supplementary Material



## Data Availability

The code used to generate the molecule ensembles for vacuum, hexane, and water environments, the crystal minimization, as well as the automated fitting code is open source and available on GitHub (https://github.com/rinikerlab/TorsionMotifTorsionAngleDistributions and https://github.com/rinikerlab/TorsionDistributionFitting). The extracted histogram data is available in the first repository in Results/torsionHistogramsAllEnvspkl as well as the newly determined fit parameters (eq 4) for the analyzed SMARTS patterns in Results/fitCoeffsAllEnvspkl. The registration of all used compounds was done using lwreg,^44^ while all additional data generated for this study was stored in additional tables. All details of the database design are provided in the TorsionMotifTorsionAngleDistribution GitHub repository.

## Notes

(S1) Hierarchical List of SMARTS patterns, (S2) Fitting Torsion Profiles with Wrapped Mirrored Gaussians, (S3) Comments on the Wasserstein Distances, (S4) Comments on Cramér-von Mises, (S5) Derivation of max­(*W*_2_) and max­(ω), (S6) Influence of the Chosen Data set, Case Example, (S7) tmTADs Across All Environments, (S8) Influence of the Energy Cutoff During Ensemble Processing, (S9) Energy Minimization in the Crystal Environment for Selected Examples (PDF)
