# Supplementary material for: Unraveling Torsional Preferences: Comparative Analysis of Torsion Motif Torsional-Angle Distributions across Different Environments
Source: J Chem Inf Model. 2025 Dec 9;65(24):13215–25. doi: 10.1021/acs.jcim.5c02311 (PMC12728936; doi:10.1021/acs.jcim.5c02311)
Supplement: Supplementary file 1 [file ci5c02311_si_001.pdf]

SUPPORTING INFORMATION
Unraveling Torsional Preferences: Comparative Analysis of Torsion Motif Torsional-Angle Distributions Across Different Environments

Jessica Braun,[a] Paul Katzberger,[a], Gregory A. Landrum,[a], Djahan Lamei,[a]
and Sereina Riniker*[a]

[a] *Department of Chemistry and Applied Biosciences, ETH Zürich, Vladimir-Prelog-Weg 2, 8093 Zürich, Switzerland. E-mail: sriniker@ethz.ch*

Contents

S1 Hierarchical List of SMARTS patterns **S2**

S2 Fitting Torsion Profiles with Wrapped Mirrored Gaussians **S2**

S3 Comments on the Wasserstein Distances **S2**

S4 Comments on Cramér-von Mises **S3**

S5 Derivation of $max(W_2)$ and $max(\omega)$ **S3**

S6 Influence of the Chosen Dataset, Case Example **S4**

S7 tmTADs Across All Environments **S6**

S8 Influence of the Energy Cutoff During Ensemble Processing **S7**

S9 Energy Minimization in the Crystal Environment for Selected Examples **S9**

S1 Hierarchical List of SMARTS patterns

The hierarchical list of SMARTS patterns as derived from the Torsion Library[1] can be found in `https://github.com/rinikerlab/TorsionMotifTorsionAngleDistributions/blob/main/Database/et4hierarchy.sql`. The README in the same GitHub repository includes the instruction on how to restore the table.

S2 Fitting Torsion Profiles with Wrapped Mirrored Gaussians

Peak Detection

The initial array containing the smoothed histogram values of the torsion distribution was rotated such that its minimum value is positioned at the first index. This transformation ensures that any peak located at 0 or 2π is not missed by scipy's `find_peaks` function, which cannot detect peaks at the boundary. After the peak detection, the resulting indices were mapped back to their original positions in the unshifted array before being returned. The approach is implemented in the fitting package's `findPeaksPeriodic` function (see `https://github.com/rinikerlab/TorsionDistributionFitting`).

S3 Comments on the Wasserstein Distances

Comparing the distributions and deciding on their closeness can be views as an optimal transport problem. The distance can be expressed as an optimal transport cost [2, 3],

$$C(\mu, \nu) = inf_{\pi \in \Pi(\mu,\nu)} \int c(x, y) d\pi(x, y), \tag{1}$$

where μ, ν are probability measures on $\mathbb{R}^d$.

Wasserstein distances express the minimal transport cost from one distribution to another [2]. The group of Wasserstein distances differs in the way they define the cost, which shows in their mathematical formulation [3],

$$W_p(\mu, \nu) = \inf_{\substack{X \sim \mu \\ Y \sim \nu}} (\mathbb{E}||X - Y||^p)^{(1/p)} \quad \text{if} p \geq 1 \tag{2}$$

In this work, we considered the W_1 and W_2 distances, which describe their cost through the Euclidean distance and the squared Euclidean distance of the distributions data points, respectively. While the W_1 distance focuses on changes in mass (shifts), the W_2 distance penalizes outliers more heavily and is also more sensitive to disturbances in the spread. Figure S1 serves as an illustration of the agreement or disagreement of the two metrics.

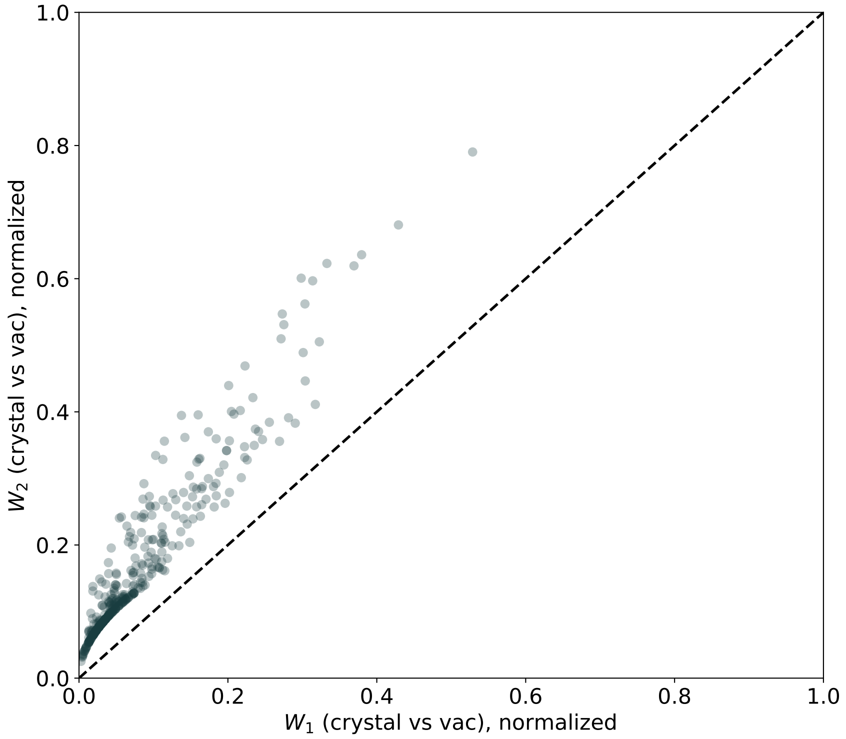

Figure S1: Comparison of the first-order Wasserstein distance W_1 and the second-order Wasserstein distance W_2 for the accumulated torsion profiles in the crystal and vacuum environments.

Furthermore, it is also important to understand the nature of the distributions in our use case. All angular data are of circular nature, which normally would prevent the usage of the standard Wasserstein distance. However, our data are also symmetric around π, as discussed in detail in the main text (section on profile extraction). Due to this property, no special implementation is required to treat circular data.

S4 Comments on Cramér-von Mises

The selection of a Cramér-von Mises derived distance metric ω is for its sensitivity to shape changes, which are of particular interest in our use case. Alternatives would be familiar statistical distances such as the Kullback-Leibler [4] or Jensen-Shannon distance [5]. However, both have properties, which make them unsuitable here: Kullback-Leibler is non-symmetric, and Jensen-Shannon does not penalize shape changes in an appropriate manner, e.g. in the examples presented in Figure 2 in the main text, it ranks case 2 (examples 3 and 4) to be the most different.

S5 Derivation of $max(W_2)$ and $max(\omega)$

In order to combine both W_2 and ω into one score τ, a normalization of both values to the same value range is needed. An empirical way to determine these values is to construct the most different case: Following the intuitive understanding of the Wasserstein distance, the most extreme situation is having to move all mass to the farthest possible place. As our data are circular, the farthest away is π, which leads to the two distribution candidates shown in Figure S2.

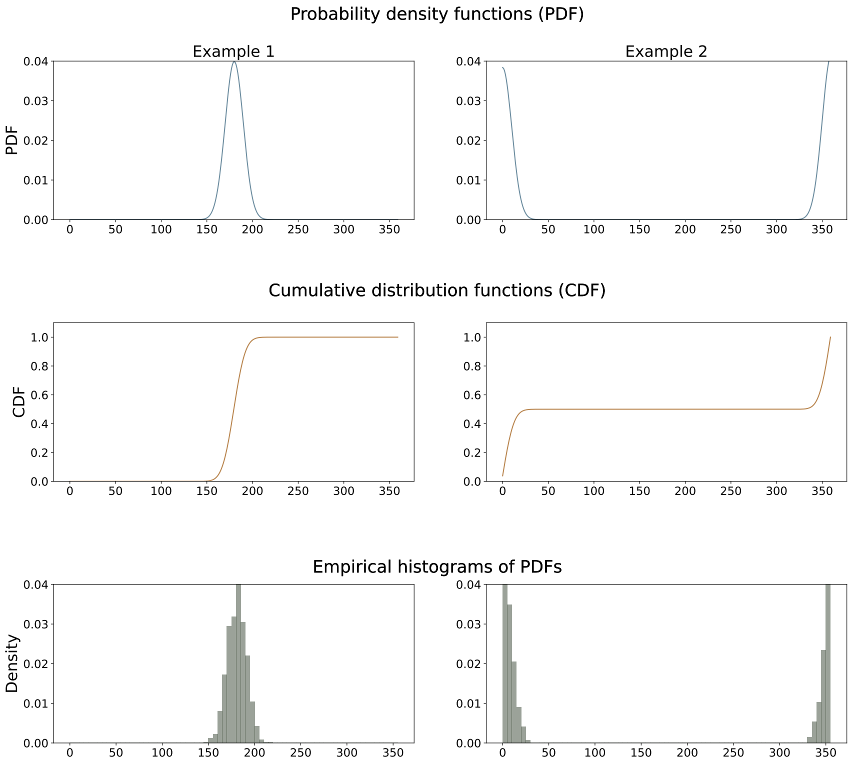

Figure S2: Case construction for the empirical determination of $max(W_2)$ and $max(\omega)$.

For examples 1 and 2 in Figure S2, the following maximum values of the two metrics were determined:

$$max(W_1) = 0.56,\ max(W_2) = 0.47,\ max(\omega) = 0.2 \tag{3}$$

S6 Influence of the Chosen Dataset, Case Example

Figure S3 serves as an example to illustrate the case where selectivity for structural isomers has a large influence on the extracted tmTAD. Given the SMARTS pattern `[!#1:1][c:2]!@;-[SX3:3][!#1:4]`, the two structural isomers shown in Figure S3 match a different total number of dihedrals, which is due to the SMARTS language recognizing a not explicitly defined bond as either single or aromatic. Therefore, the double bonded oxygen is not matched in the non-zwitterionic form, which leads to the absence of two peaks in the accumulated torsion profile in Figure S4.

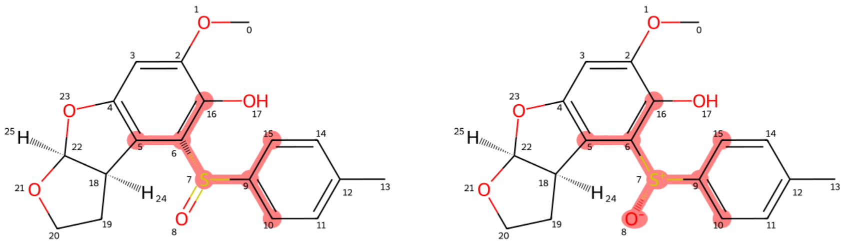

Figure S3: Example of structural isomers with the zwitterionic form presented on the right hand side. All dihedrals matching the SMARTS `[!#1:1][c:2]!@;-[SX3:3][!#1:4]` are highlighted in red.

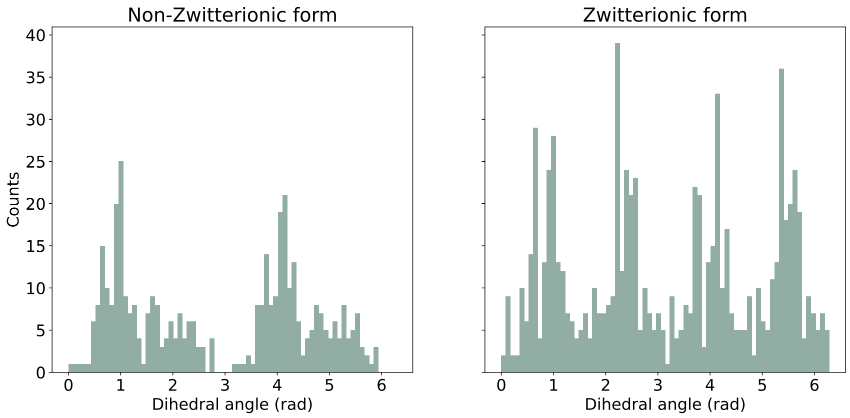

Figure S4: Torsion profiles for the example in Figure S3. The two absent peaks in the left panel are due to the non-matching of the SMARTS pattern to the double bonded oxygen in the non-zwitterionic form.

Both the DASH and CSD datasets have representatives of the two forms present, but the ratio of their occurrence is different. The ratio between the zwitterionic:non-zwitterionic is 34:1450 in the CSD, whereas in the DASH dataset, it is 941:537. Figure S5 shows the resulting distributions for the DASH and CSD datasets for the SMARTS pattern in question. The different ratios mean that in the case of the DASH dataset, the distribution is much more uniform across all torsion-angle values with the peaks at the positions from the zwitterionic form dominating.

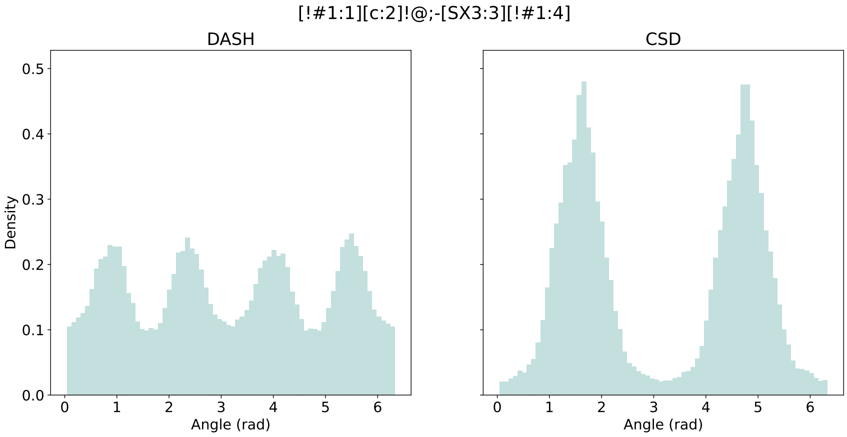

Figure S5: Torsion distributions for the SMARTS pattern `[!#1:1][c:2]!@;-[SX3:3][!#1:4]` for the two datasets DASH (left) and CSD (right). Extractions were done with respect to the SMARTS hierarchy.

S7 tmTADs Across All Environments

Figure S6 shows the distributions of the W_2 and ω values for the six pairwise comparisons.

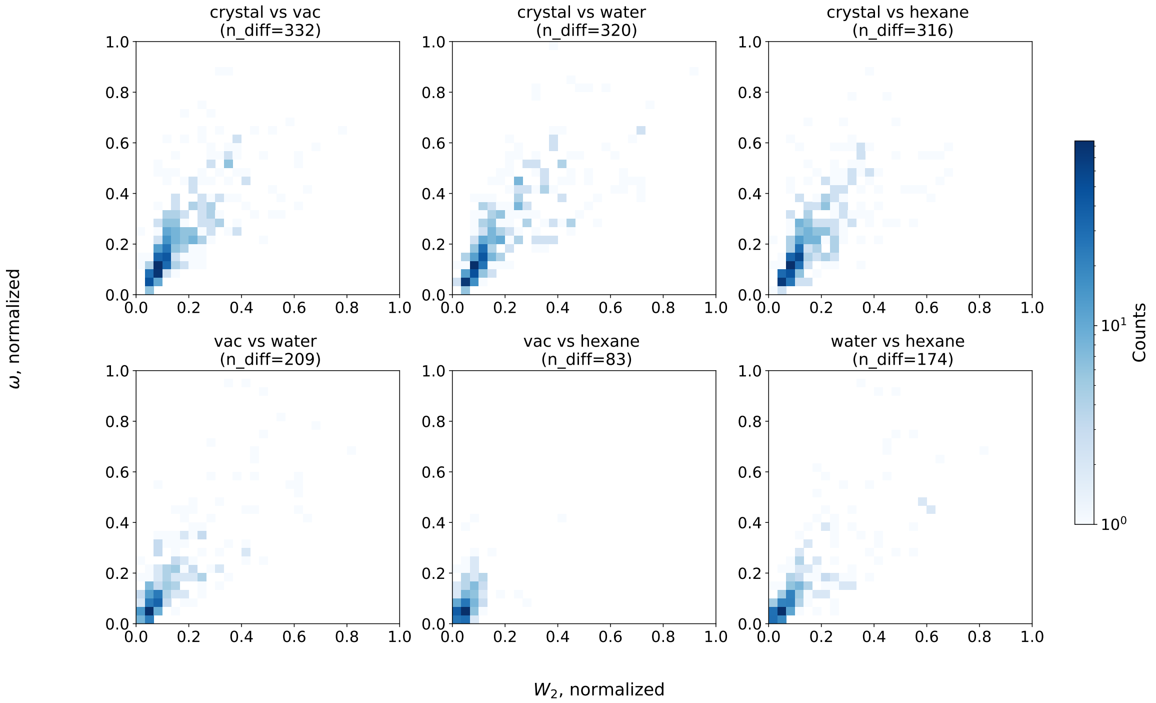

Figure S6: 2D histograms (density heatmap) of the normalized statistical distances W_2 and ω for the six pairwise comparisons between environments. In parentheses is the number of significantly different distributions for each group as determined by the τ threshold. Top: Comparison of the crystal environment with the three others. Bottom: Comparison of the three computed environments (vacuum, hexane, water).

S8 Influence of the Energy Cutoff During Ensemble Processing

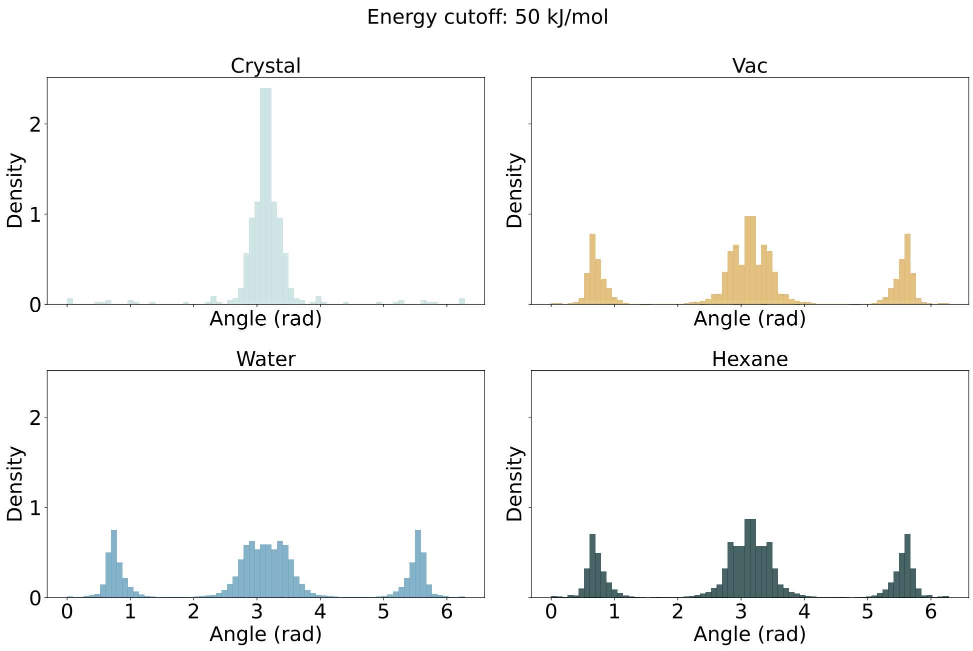

Figure S7: Torsion distributions for SMARTS pattern `[CX3H0:1]=[CX3H0:2]!@;-[CX3:3]=[CX3:4]` in selected environments with the extractions using all conformers associated with an energy less than 50 kJ/mol.

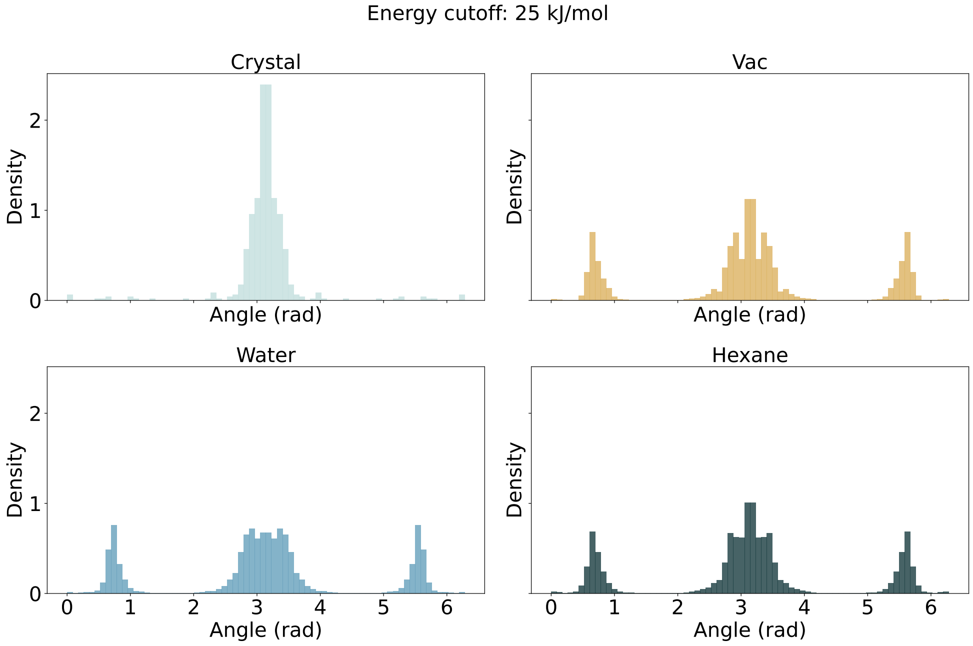

Figure S8: Torsion distributions for SMARTS pattern `[CX3H0:1]=[CX3H0:2]!@;-[CX3:3]=[CX3:4]` in selected environments with the extractions using all conformers associated with an energy less than 25 kJ/mol.

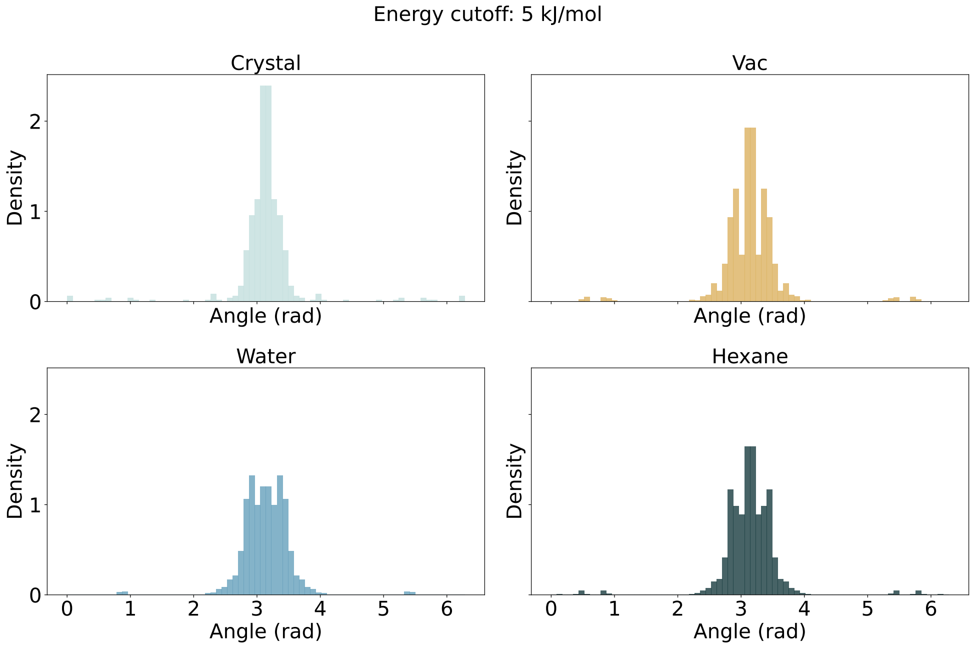

Figure S9: Torsion distributions for SMARTS pattern `[CX3H0:1]=[CX3H0:2]!@;-[CX3:3]=[CX3:4]` in selected environments with the extractions using all conformers associated with an energy less than 5 kJ/mol.

S9 Energy Minimization in the Crystal Environment for Selected Examples

For selected cases, the influence of the force field Openff-2.0.0 [6] was assessed to try to entangle effects from force-field biases versus the environment. As each CSD entry includes the information of the space group and the unit cell parameters, the asymmetric unit and symmetry equivalent positions, the (asymmetric) unit cell can be constructed. Subsequently, an energy minimization with OpenMM 8.1.2 [7] was performed at 300 K.

Note that this analysis is not a systematic study but a set of illustrative examples demonstrating that energy minimization alone is inconclusive for assessing force-field effects in the crystal, because minimization preserves the original packing of small-molecule crystals.

Figure S10 shows the overlay between the original unit cell and the energy-minimized unit cells for the two examples studied in the main text. Only minimal changes can be observed, which does not mean that the crystal conformation is the global minimum of the force field.

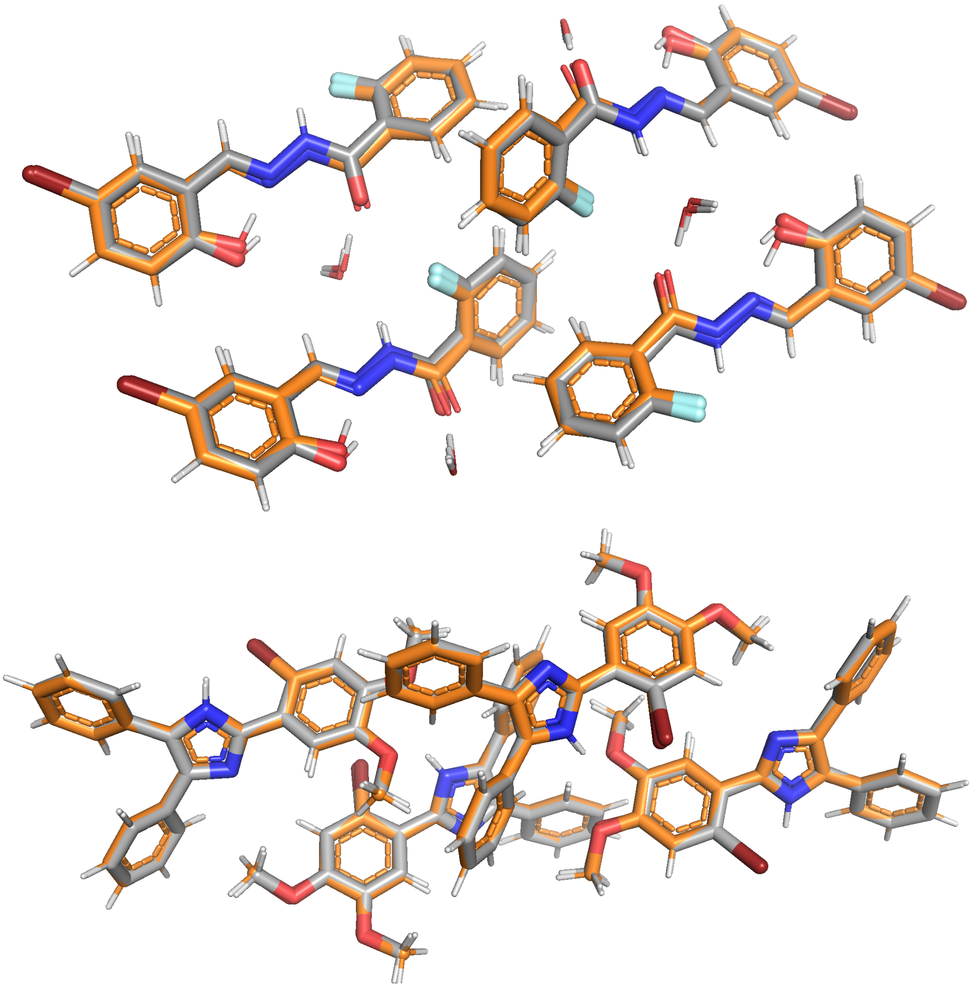

Figure S10: Overlay of the original unit cell as deposited in the CSD (grey carbons) and the energy-minimized unit cell (orange carbons) for examples ABOQOZ (top) and ABAGUJ (bottom).

Analysis of Two Selected Torsion Motifs

To illustrate this point further, two additional SMARTS patterns (A) `[CX3H0:1]=[CX3H0:2]!@;-[CX3:3]=[CX3:4]` and (B) `[cH:1][c:2]([cH])!@;-[O:3][$(C([F])([F])[F]):4]` were selected as they show noticeable differences in their crystal tmTAD compared to the other three environment tmTADs. For both patterns, we provide the tmTADS and the comparison between the nearest local energy minimum modeled by the force field and the original unit cell two example molecules that contain the pattern.

Pattern A

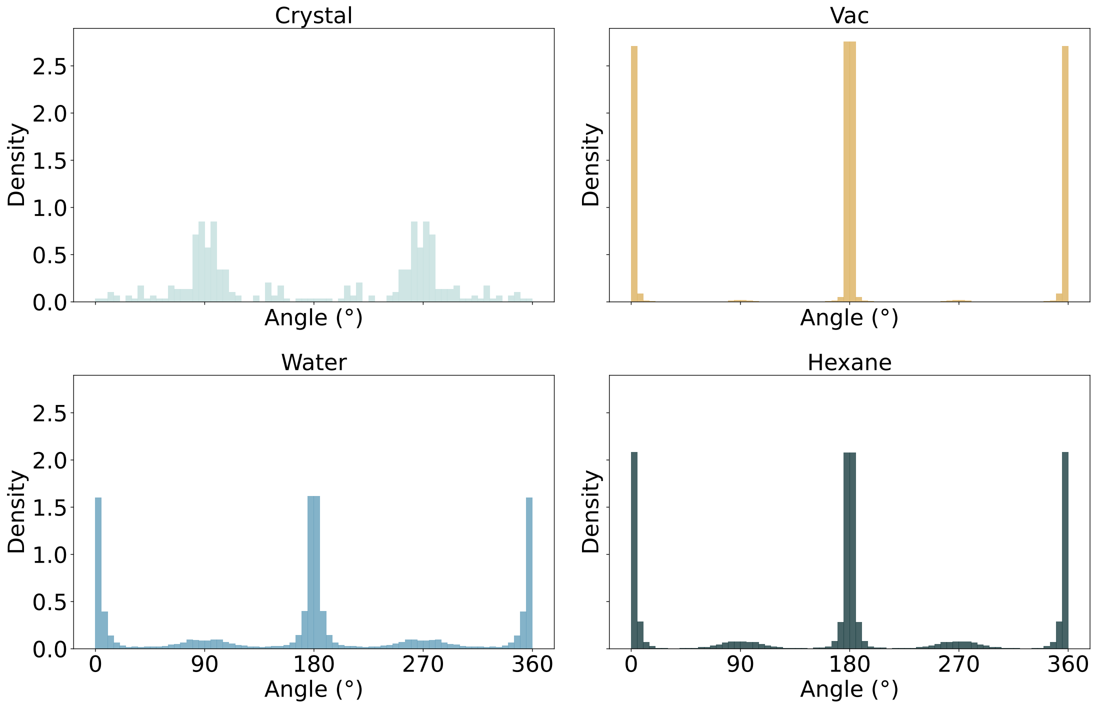

Figure S11: tmTADs for SMARTS pattern (A) `[cH:1][c:2]([cH])!@;-[O:3][$(C([F])([F])[F]):4]` in the four environments.

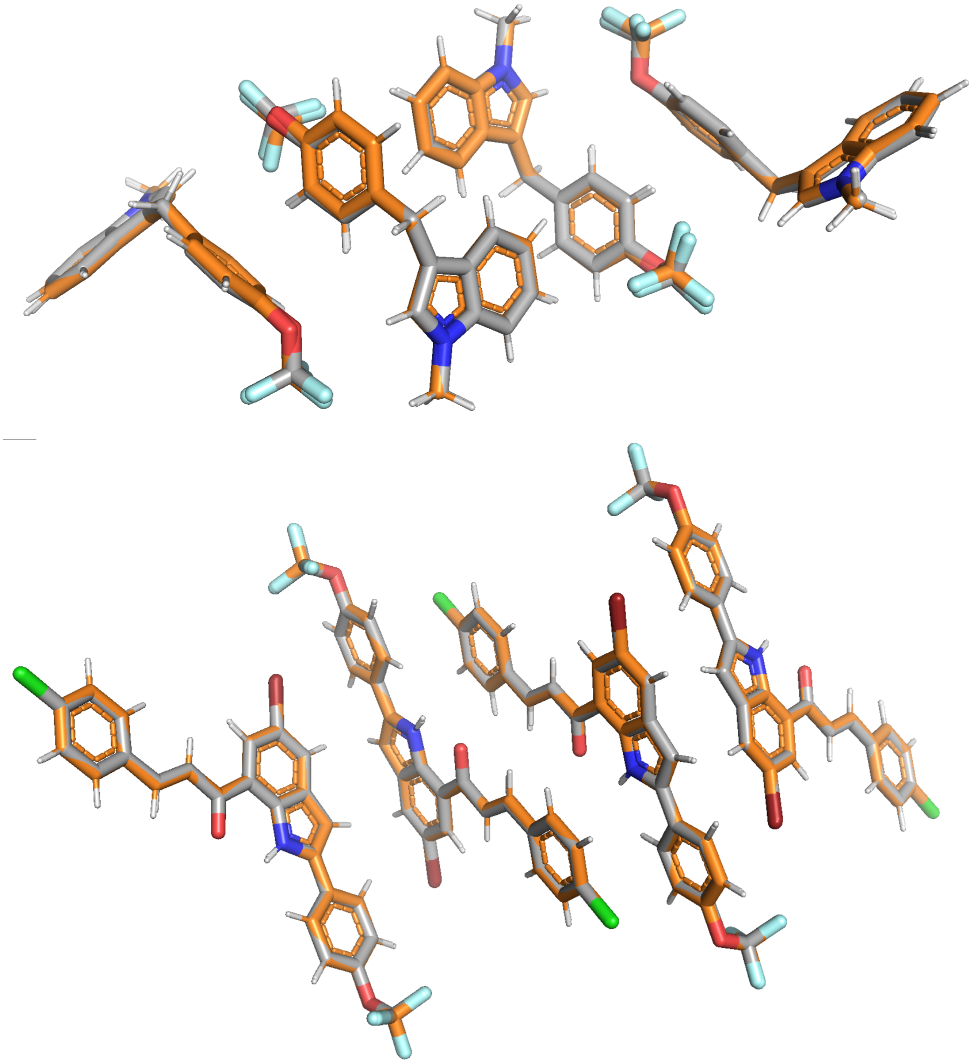

Figure S12: Overlay of the original unit cell as deposited in the CSD (grey carbons) and the energy-minimized unit cell (orange carbons) for examples MOJYAP (top) and EROXIU (bottom).

Pattern B

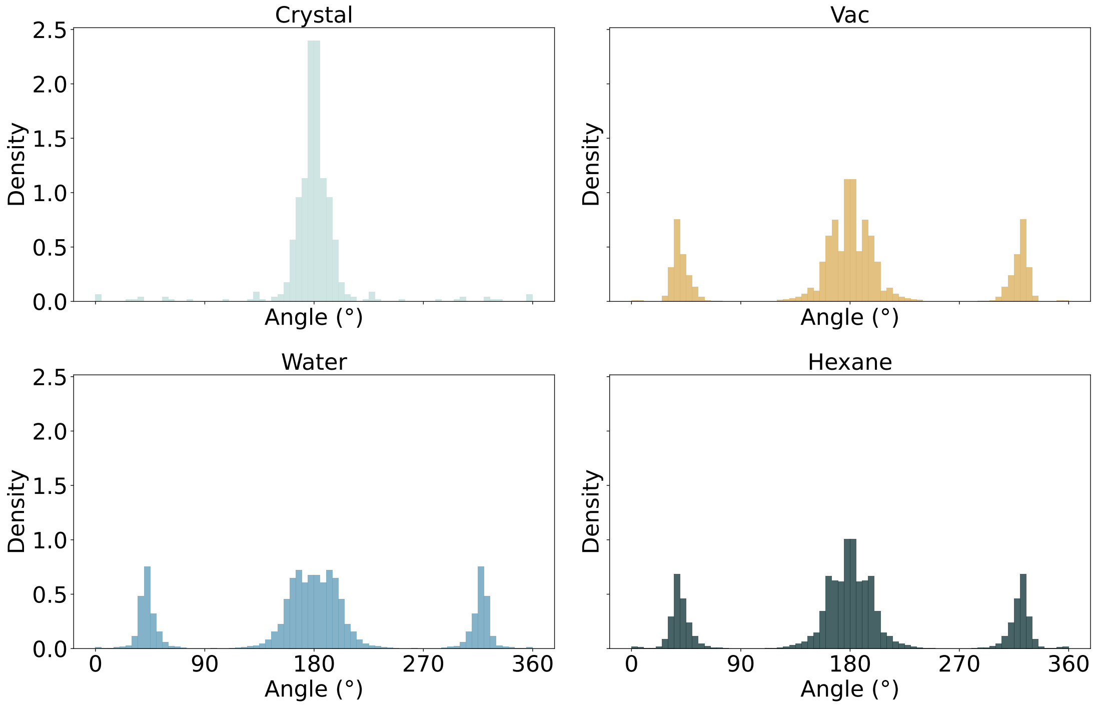

Figure S13: tmTADs for SMARTS pattern (B) `[CX3H0:1]=[CX3H0:2]!@;-[CX3:3]=[CX3:4]` in the four environments.

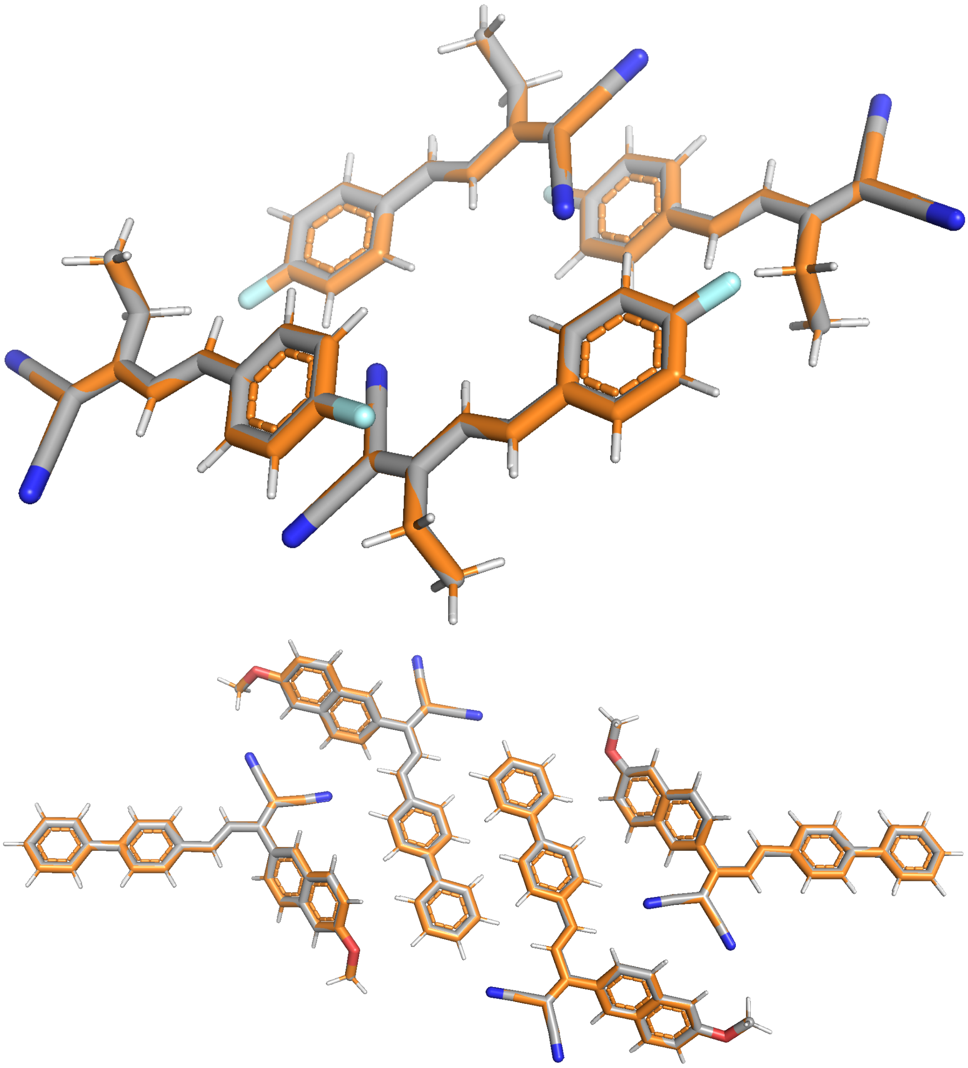

Figure S14: Overlay of the original unit cell as deposited in the CSD (grey carbons) and the energy-minimized unit cell (orange carbons) for examples ABAKIZ (top) and AJUJIB (bottom).

References

[1] Penner, P.; Guba, W.; Schmidt, R.; Meyder, A.; Stahl, M.; Rarey, M. The Torsion Library: Semi-automated Improvement of Torsion Rules with SMARTScompare. *J. Chem. Inf. Model.* **2022**, *62*, 1644–1653.

[2] Villani, C. *Optimal Transport*; Grundlehren der mathematischen Wissenschaften; Springer Berlin Heidelberg, Vol. 338; pp 93–111.

[3] Panaretos, V. M.; Zemel, Y. Statistical Aspects of Wasserstein Distances. *Annu. Rev. Stat. Appl.* **2019**, *6*, 405–431.

[4] Kullback, S.; Leibler, R. A. On Information and Sufficiency. *Ann. Math. Statist.* **1951**, *22*, 79–86.

[5] Lin, J. Divergence measures based on the Shannon entropy. *IEEE Trans. Inform. Theory* **1991**, *37*, 145–151.

[6] Boothroyd, S. et al. Development and Benchmarking of Open Force Field 2.0.0: The Sage Small Molecule Force Field. *J. Chem. Theory Comput.* **2023**, *19*, 3251–3275.

[7] Eastman, P. et al. OpenMM 8: Molecular Dynamics Simulation with Machine Learning Potentials. *J. Phys. Chem. B* **2024**, *128*, 109–116.
